# Whistleblowing in financial practice: a review of processes and impacts

**DOI:** 10.3389/fsoc.2026.1725253

**Published:** 2026-04-01

**Authors:** Najla Al-Thani, Steven Wright

**Affiliations:** College of Humanities and Social Sciences, Hamad bin Khalifa University, Qatar Foundation, Doha, Qatar

**Keywords:** financial accountability, fraud deterrence, organizational culture, regulatory compliance, whistleblower protection, whistleblowing

## Abstract

Corporate fraud detection frequently relies on whistleblowing as a primary deterrent mechanism. The financial services sector is widely recognized as carrying significant fraud risk; however, persistent barriers to anonymous whistleblowing remain, including cultural obstacles, insufficient protections, and varying enforcement mechanisms across jurisdictions. Although the importance of whistleblowing is increasingly acknowledged and practiced, evidence remains limited and uneven regarding the influence of organizational and cultural contexts on whistleblowing behavior, particularly in emerging economies with developing regulatory environments. This critical review provides an integrative summary of the literature to develop a multi-theoretical model describing whistleblowing processes, outcomes, and cross-national policy effectiveness within financial services. The review employs a theory-informed narrative synthesis within an integrative review design, analyzing publications from 2000 to 2025 using relevance-based selection and a five-theory analytic framework (moral judgement, power dependence, expectancy, institutional, and network governance). Evidence was synthesized within each theoretical lens and then integrated to construct a multi-level conceptual framework. The synthesis indicates that whistleblower outcomes are shaped by social processes involving negotiations among individual moral characteristics, organizational power dynamics, institutional barriers, and regulatory networks. Despite legal reforms, whistleblowers remain vulnerable in organizations with weak cultures. Elevated retaliation risk highlights tensions between protective policies and market pressures. Comparative analysis identifies robust regimes under Sarbanes–Oxley, Dodd–Frank, and the EU directive, while emerging economies often lack effective enforcement and cultural support for disclosure. Strengthening protection-oriented policies in these jurisdictions and embedding whistleblowing within Basel operational risk frameworks is recommended.

## 1 Introduction

Despite this importance, whistleblowers often face serious consequences, including retaliation, job loss, and reputational harm (Kenny et al., 2019; Park et al., 2020). These risks are especially paramount in jurisdictions with weak institutional protections, hierarchical organizational cultures, and limited regulatory enforcement capacity (Latan et al., 2023; Abazi, 2020). In such contexts, the deterrent effect of whistleblowing is undermined, allowing misconduct to persist and eroding organizational accountability. This is consistent with evidence that higher expected retaliation costs suppress reporting, while stronger protections/incentives increase it (Heese and Pérez-Cavazos, 2021; Berger and Lee, 2022). While legal reforms have been introduced in many jurisdictions, enforcement remains inconsistent, and organizational responses are often inadequate (Abazi, 2020; ESMA, 2024) (see Section 5.4 for illustrative cases).

Fraud detection in practice relies heavily on reporting channels, with occupational-fraud evidence consistently identifying tips as a leading detection mechanism (ACFE, 2024). Yet reporting remains constrained by perceived retaliation risk and uneven protection: cross-national evidence indicates that reprisals are common and that reporting behavior varies by sector, organizational hierarchy, and the credibility of enforcement (OECD, 2021; Chalouat et al., 2019). These dynamics are likely to be amplified in emerging economies, where enforcement visibility and cultural support for disclosure may be weaker (FATF, 2007). Fraud-detection evidence highlights the importance of reporting channels in uncovering misconduct, yet perceived retaliation risk and uneven protection can suppress disclosure, especially when enforcement is weak.

Despite recent research, the evidence base remains concentrated in the US and EU, and theoretical work is dispersed across moral, incentive, power, and institutional perspectives. A gap between law and practice limits the effectiveness of protections, particularly where confidential intake, anti-retaliation measures, and remediation are insufficient. This review provides an in-depth analysis of whistleblowing in the financial sector, with a focus on banking. It examines processes, stakeholders, and outcomes; explores the reasons individuals report or remain silent; and analyzes the organizational and cultural factors influencing these decisions, highlighting the need for stronger protections and support to enable whistleblowers to report without personal or professional harm (Park et al., 2020; Heese and Pérez-Cavazos, 2021). The theory-informed integrative review pursues three synthesis objectives: (1) to synthesize evidence on whistleblowing in financial services, emphasizing reporting processes, key actors, and commonly discussed outcomes; (2) to interpret and organize the literature using five complementary theoretical lenses (moral judgement, power-dependence, expectancy, institutional, and network governance), integrating insights into a multi-level conceptual framework; and (3) to identify cross-jurisdictional implications by contrasting how protection and enforcement contexts shape disclosure conditions, with particular attention to policy and governance considerations in emerging economies.

## 2 Background and theoretical framework

### 2.1 Introduction to whistleblowing

Near and Miceli (1985) definition of whistleblowing as an insider's disclosure of deliberate malfeasance was helpful but restrictive. Jubb (1999) argued that this definition was formulated in the context of corporate/private organizations, which implicitly assumes an employer-employee relationship between the whistleblower and the organization. Whistleblowing has been defined as a voluntary, public-interest–oriented disclosure of significant wrongdoing to an authority able to investigate and facilitate corrective action (Maria, 1995) as cited in Martin and Rifkin (2004). Similarly, Transparency International (2013) described whistleblowing as the disclosure (to individuals or entities believed to be able to effect action) of information related to corrupt, illegal, fraudulent or hazardous activities being committed in or by organizations in the public or private sector—activities which are of concern or pose threats to the public interest (Transparency International, 2013).

There is some consensus among researchers that disclosures made through whistleblowing most commonly involve unethical behavior, abuse of authority, corruption, embezzlement, mismanagement, waste of funds, and professional misconduct (Gobert and Punch, 2000; Quayle, 2021). The roots of whistleblowing can be traced to various historical instances, including the American Revolution and the Civil War era (Olesen, 2022). However, its significance in more recent times can be attributed to a few key events. One notable catalyst is the disclosure of the Pentagon Papers by Daniel Ellsberg, a former military analyst who revealed classified documents that exposed the US government's misleading portrayal of the Vietnam War (Di Salvo, 2016; Kitrosser, 2011). Similarly, the Watergate scandal exposed political wrongdoing at the highest levels of the US government (Di Salvo, 2020; Li, 2014). In the corporate sphere, the Enron scandal, which involved large-scale corporate fraud and misconduct, was brought to light only through whistleblowing (Fanto, 2004). In recent years, high-profile cases involving Edward Snowden, Chelsea Manning, and Julian Assange have highlighted the role of whistleblowing in exposing corruption, fraud, abuse of power, and other forms of wrongdoing within the context of public governance (Di Salvo, 2016; Touchton et al., 2020).

Many researchers agree that several factors may contribute to an individual's decision to engage in whistleblowing and thereby disclose or expose illegalities or wrongdoing within organizations or among those in power (Djamal et al., 2019; Ikbal, 2017; Rustiarini and Sunarsih, 2017). Understanding the reasons may lead to a deeper theoretical understanding of whistleblowing. Insofar as whistleblowing ensures accountability and promotes the public interest regarding the activities of government, whistleblowers are usually understood as ‘insiders' who are privy to valuable information that the public cannot obtain or access from oversight systems, information regarding activities which are illegal and inimical to society's interest (Dworkin and Baucus, 1998; Choo et al., 2019; Jubb, 2000). Similarly, whistleblowing has been described as a fundamental tool, a safeguard against corruption that enables the exposure of all forms of financial misconduct—including fraud, theft, embezzlement, and misappropriation (Carr and Lewis, 2010; Gibbs, 2020). Whistleblowing tends to enhance the integrity and financial transparency of institutions responsible for governance and the management of public resources. Furthermore, in societies with robust moral and ethical standards, especially within public administration, individuals may feel morally or ethically bound to blow the whistle on wrongdoing (Bok, 1989). In the case of Edward Snowden, for example, exposing widespread US government surveillance practices—thereby disclosing to the public potential violations of their privacy and civil liberties—was, for the whistleblower, a moral duty (Ceva and Bocchiola, 2020; Delmas, 2015; Scheuerman, 2014).

Several authors have sought to address concerns that cast whistleblowing as a risk not worth taking. Kumar and Santoro (2017) argued that whistleblowing is justified when disclosures are made with proper intent and fulfill specific communicative constraints to address issues of public interest. They proposed a tripartite communicative constraint—informativeness, truthfulness, and evidence—which, if established, would justify whistleblowing. As they argued, whistleblowing should be encouraged when disclosures related to the public interest are made properly, with the right intent, in compliance with clear procedures, and through a protective system that insulates whistleblowers from any immediate or future harm (Kumar and Santoro, 2017). There is recognition of the need for countries to implement robust legislative frameworks, to adopt the best international practices that protect whistleblowers, and thereby to address the impediments against reporting wrongdoing (Chalouat et al., 2019; Devine and Walden, 2013; Gibbs, 2020; Mechtenberg et al., 2020; Sawyer et al., 2010; Schultz and Harutyunyan, 2015; European Parliament and Council of the European Union, 2019; OECD, 2011).

Some scholars define whistleblowing “effectiveness” using outcome-based criteria. Near and Miceli (1995) define effective whistleblowing in terms of whether the reported wrongdoing is stopped, at least partly due to the disclosure, within a reasonable time frame (Near and Miceli, 1985). Other work frames effectiveness in terms of whether the whistleblower's aims are achieved and whether the disclosure is acted upon by relevant audiences. In a related organizational-outcome framing, Dworkin and Baucus (1998) treat whistleblowing as effective when it triggers an organizational investigation and/or corrective action (e.g., changes to policies or procedures, or elimination of wrongdoing).

Some researchers seeking to define whistleblowing's effectiveness have proposed a threshold to assess its effectiveness. Near and Miceli (1995) propose assessing effectiveness by the extent to which the questioned or wrongful practice is terminated, at least partly because of whistleblowing, within a reasonable time frame (Near and Miceli, 1995). Others frame effectiveness in terms of whether whistleblowers achieved their intended impact and whether relevant audiences responded to their warnings (Elliston et al., 1985). In a related outcome-oriented approach, Dworkin and Baucus (1998) describe effectiveness as occurring when an organization initiates an investigation into the allegations (voluntarily or under external requirement) and/or takes corrective steps such as changing policies, procedures, or eliminating wrongdoing (Dworkin and Baucus, 1998).

### 2.2 Theoretical foundations

Various theories, including moral judgment, power-dependence, and expectancy theories, may explain whistleblowing. The following theories are introduced below in relation to their connection to whistleblowing.

#### 2.2.1 Moral judgement theory

**Figure 1:**
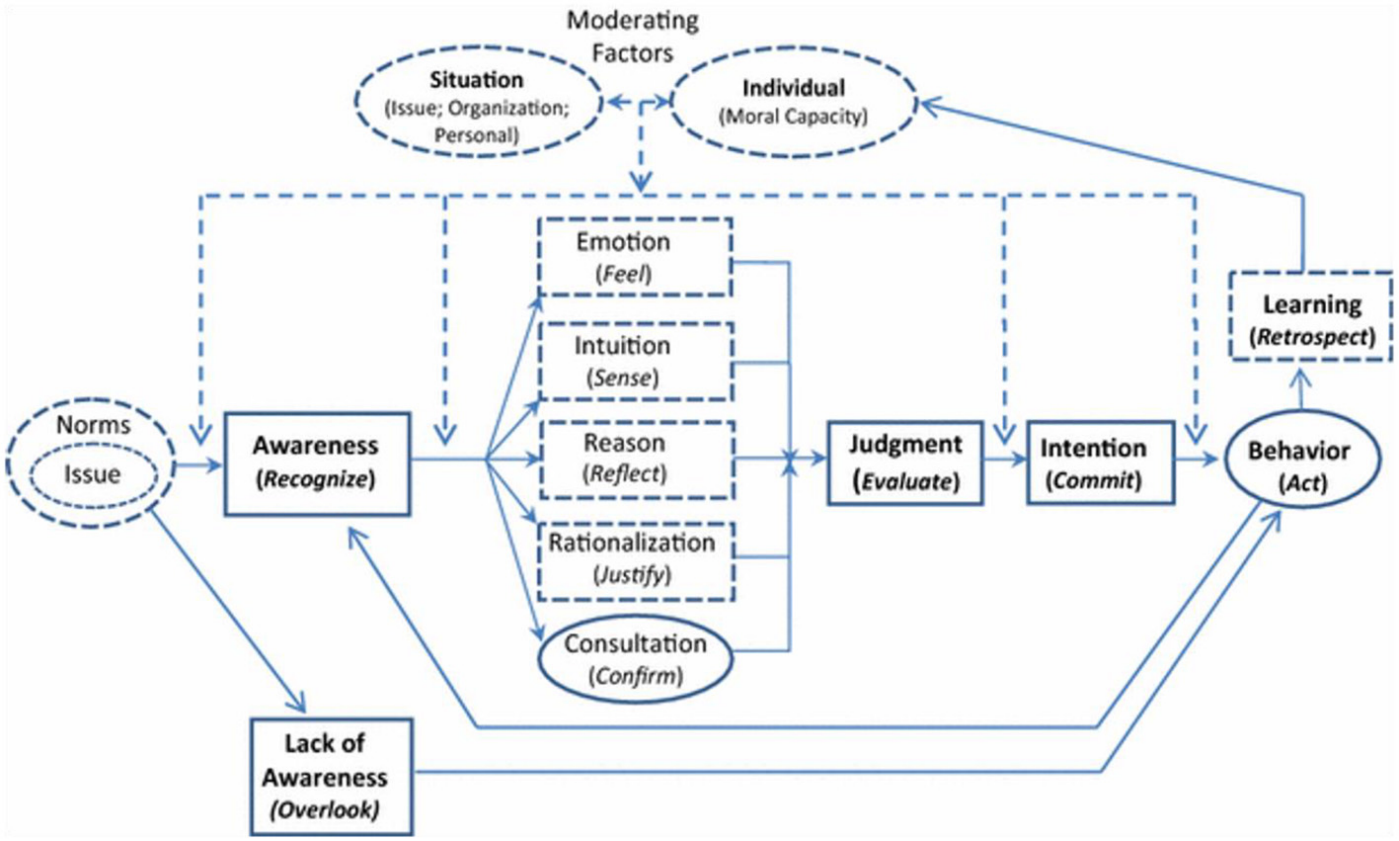
An integrated perspective of ethical decision-making (Schwartz, 2016). Used with permission.

Moral judgment theory may explain whistleblowing activities that contradict the cost-benefit analysis, often highlighting various perspectives on whistleblowing. As Watts and Buckley (2017) observed, the cost-benefit perspective fails to explain why some people blow the whistle even when they face affective discomfort, psychological trauma, and a low likelihood of personal benefit. On a different approach, moral judgment theory suggests that whistleblowing arises from moral concern (Watts and Buckley, 2017), which is defined as an individual's concern for others' well-being or for what is right or wrong (Dungan et al., 2019). Dungan et al. (2019) found that moral concern is associated with whistleblowing decisions even after controlling for organizational and situational factors (Dungan et al., 2019). Schwartz (2016) suggested that moral judgement could arise from the interaction between rational factors associated with logic and non-rational factors linked with intuition and emotion. Rational effects belong to the logical assessment of weighing various moral standards to reach a decision that resolves conflicts among competing moral principles (Schwartz, 2016). Non-rational effects arise from intuitions and emotions, which initiate moral judgments, while logical explanations later support retrospective rationalizations (Schwartz, 2016). Figure 1 illustrates the interactions of various rational and non-rational factors that influence moral judgment after an individual becomes aware of a moral issue.

#### 2.2.2 Power-dependence theory

Power-dependence theory holds that power processes are integral to whistleblowing. Under this theory, whistleblowing is perceived as an influence process in which stakeholders seek to gain power over others (Near et al., 1993). The theory suggests that a whistleblower's action is intended to exert power over the organization by persuading the dominant coalition to act against the members targeted by the power exertion (Near et al., 1993). Taking action, the dominant coalition may accept the whistleblower's exertion of power (thus acting against the wrongdoing) or it may fail to accept the whistleblower's power [thus failing to remedy the wrongdoing; Near et al. (1993)]. Moreover, the dominant coalition may try to enforce its preferred balance of power by retaliating against the whistleblower (Near et al., 1993). These explanations based on power theory are closely related to the resource-dependence perspective, in which the exertion of power—whether by the whistleblower or the organization—is intended to reduce one party's dependence on the counterparty (Near et al., 1993). This theory may explain how organizations respond to whistleblowing events, particularly when retaliation against whistleblowers occurs.

#### 2.2.3 Expectancy theory

Expectancy theory has developed in the context of employee motivation but could also be applied to the whistleblowing process. The theory refers to concepts developed by various authors, most notably Victor Vroom, Lyman Porter, and Edward Lawler (Miner, 2015). It suggests that individuals are motivated to act based on their own evaluations of the potential to achieve a desired performance (expectancy). They may believe that achieving the desired performance will lead to desired outcomes (instrumentality), and that the outcomes would have personal value (Miner, 2015). An individual may be incentivized to blow the whistle if they believe doing so would end the wrongdoing or punish the wrongdoer (expectancy). Similarly, they may be incentivized if they believe their actions would lead to positive outcomes, such as a personally valuable reward (Potipiroon, 2024). This theory supports both the existence of mechanisms to avoid retaliation against whistleblowers and the use of rewards to encourage whistleblowing.

#### 2.2.4 Institutional theory

Institutional theory explains how formal rules, informal norms, and societal expectations shape organizational behavior (Scott, 2001). Applied to whistleblowing, it highlights how pressures from regulators, cultural values, and professional standards influence whether organizations establish credible reporting mechanisms (Okafor et al., 2020). Institutional theory sheds light on cross-national differences in whistleblowing effectiveness in the financial industry. Financial institutions operate within nested institutional settings, including regulatory mandates from central banks, securities regulators, and prudential regulators; professional expectations from banking associations; and social expectations regarding corporate social responsibility (De Souza and De Souza, 2024). When these institutional forces align to require transparency and protection (as in jurisdictions with strict enforcement of Sarbanes-Oxley or the EU Whistleblower Directive), whistleblowing processes are incorporated into the organization (Gerdemann and Colneric, 2020). Where regulatory enforcement is weak or hierarchy norms discourage challenging authority, formal procedures are in place with little to no impact (Okafor et al., 2020; Krügel and Uhl, 2023). In contexts such as Qatar, differences in how reporting systems are adopted and communicated within organizations may shape whistleblowing practices, even when formal legal provisions are established.

#### 2.2.5 Network governance and accountability dimensions

Whistleblowing in the financial sector also operates across networks of regulators, firms, and international institutions. Network governance perspectives (Provan and Kenis, 2008) emphasize the interdependence of actors in addressing misconduct, while Mulgan (2003) dimensions of accountability illustrate how responsibility extends beyond organizations to wider systems of oversight. These frameworks explain why cross-border standards such as Basel or EU directives matter: they create shared accountability mechanisms that can strengthen whistleblower protections in otherwise weak regulatory environments (European Union, 2019). For example, while multinational banks operate in multiple jurisdictions, regulations such as the US Foreign Corrupt Practices Act and Dodd-Frank have extraterritorial reach, exerting network pressures for compliance that transcend national boundaries (U.S. Department of Justice et al., 2020). Likewise, Basel operational risk requirements require assessment of fraud risk, creating institutional incentives to establish whistleblower procedures in places with limited domestic enforcement (Basel Committee on Banking Supervision, 2025).

#### 2.2.6 Integrative framework: complementary roles and analytical mapping of the theories

This review applies five theories as complementary analytical lenses to organize and interpret evidence on whistleblowing in financial services. The selection of theories is based on relevance rather than exhaustiveness. The aim is not to encompass all theoretical approaches in the whistleblowing literature, but to employ a defined set of lenses that collectively highlight (i) individual ethical appraisal and motivation, (ii) organizational power dynamics and responses, and (iii) institutional and network conditions influencing protection, escalation, and outcomes.

At the individual level, moral judgment theory provides a framework for interpreting whistleblowing as an ethically grounded response to perceived wrongdoing, emphasizing the roles of moral concern and ethical decision-making processes (Watts and Buckley, 2017; Dungan et al., 2019; Schwartz, 2016). Expectancy theory complements this perspective by conceptualizing reporting as a motivated choice shaped by perceived expectancy, instrumentality, and valence, specifically whether reporting is anticipated to result in meaningful outcomes and whether those outcomes are valued (Miner, 2015; Potipiroon, 2024). These two theoretical lenses are applied to interpret variation in reporting intentions and choices under differing perceived costs, protections, and incentives.

At the organizational level, power–dependence theory conceptualizes whistleblowing as an influence process involving dominant coalitions and contested power relations. This perspective facilitates interpretation of organizational responses such as remediation, inaction, suppression, or retaliation (Near et al., 1993). The synthesis employs this lens to examine how internal power structures may mediate the pathway from disclosure to outcomes.

At the system level, institutional theory enables analysis of how formal rules, norms, and enforcement credibility influence the implementation, trust, and practical use of reporting mechanisms (Okafor et al., 2020). Network governance and accountability perspectives further extend this analysis to multi-actor oversight arrangements in financial services, where responsibilities are distributed among firms, regulators, and cross-border governance systems (Provan and Kenis, 2008). Additionally, transnational compliance and supervisory frameworks, such as Basel Framework requirements, shape governance expectations in banking (Basel Committee on Banking Supervision, 2025).

Table 1 summarizes how the five theoretical perspectives introduced in Section 2 are used as complementary analytical lenses in this review. It maps each theory to its primary level of analysis and the whistleblowing process components it most directly informs, and it indicates how each lens contributes to the theory-guided synthesis developed in Section 5.

**Table 1:** Analytical mapping of the five-theory framework to levels of analysis, process focus, and synthesis contribution.

Theory	Level	Process focus	Core constructs	Contribution to synthesis
Moral judgement theory	Individual	Ethical appraisal; disclosure decision	Moral concern; rational vs. non-rational moral judgement (intuition/emotion); ethical decision processes	Interprets how ethical appraisal and moral concern may shape disclosure intentions under salient personal costs
Expectancy theory	Individual	Reporting choice; perceived consequences	Expectancy; instrumentality; outcome value	Interprets how perceived likelihood and value of reporting outcomes may shape reporting decisions
Power–dependence theory	Organizational	Organizational response; retaliation dynamics	Influence process; dominant coalition response; retaliation; dependence balance	Interprets how power relations and dominant coalition responses may shape remediation, inaction, or retaliation
Institutional theory	System/jurisdiction	Implementation; enforcement context	Formal rules; informal norms; societal expectations; enforcement credibility; institutional pressures	Interprets why protections and reporting systems may function differently across institutional environments
Network governance and accountability	System/transnational	External escalation; oversight arrangements	Actor interdependence; oversight networks; accountability dimensions; cross-border compliance pressures	Interprets how distributed oversight and transnational pressures may shape escalation pathways and accountability expectations

## 3 Whistleblowing process

### 3.1 Process-oriented view

**Figure 2:**
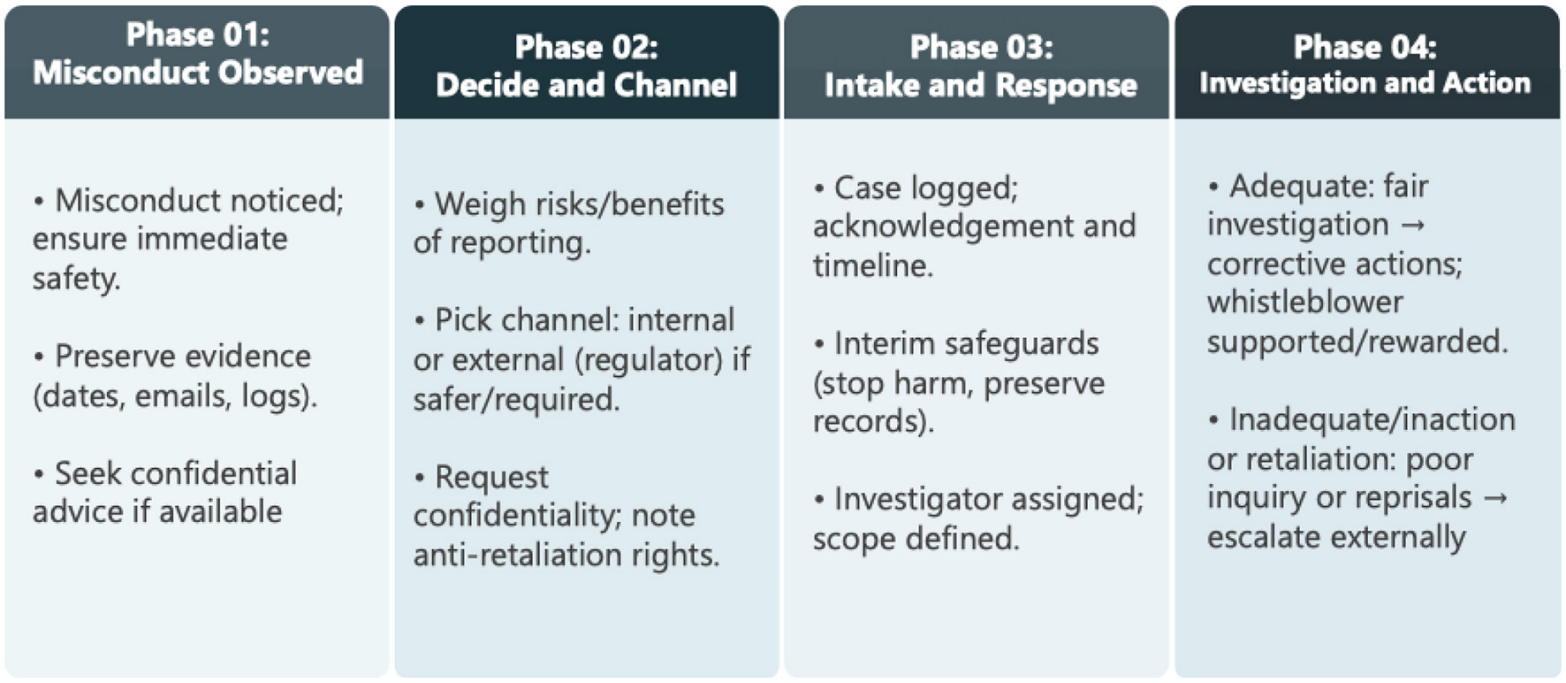
Summary of the whistleblowing process. It shows that most whistleblowing begins internally, but the process often stalls when organizations respond inadequately or retaliate. This dynamic explains why many whistleblowers either remain silent or escalate to external regulators and the media.

Brennan (2020) delineated whistleblowing as a process involving four steps and three leading actors: The whistleblower first observes wrongdoing, then willingly reports on the individuals behind it; these individuals, responsible for the organization's response, demonstrate action or inaction; target organizations of the alleged misbehaviours then call for another review covering the organization's reaction. Whistleblowing, an important element of internal control systems, helps enterprises minimize financial losses while protecting their reputations and avoiding regulatory oversight (Brennan, 2020). Lee and Xiao (2018) portray an individual who observes misconduct and faces the question of whether to blow the whistle, either within the system in which they work or externally to regulators and the media. While whistleblowers usually choose internal reporting, organizations' responses vary from favorable (e.g., launching thorough investigations and protecting whistleblowers) to unfavorable (e.g., mounting inadequate investigations, failing to correct their misbehavior, or even seeking retribution against the whistleblower (Lee and Xiao, 2018). This process-oriented perspective of whistleblowing is illustrated in Figure 2.

There are examples of adverse outcomes for whistleblowers, such as reports going unheeded or resulting in retaliation against them (European Union, 2019). In these cases, external whistleblowing might be preferable; the potential whistleblower may decide to remain quiet, ending the whistleblowing process at step one.

### 3.2 Key stakeholders

#### 3.2.1 Whistleblowers

In organizations, whistleblowing is a critical means of maintaining transparency and accountability. Whistleblowers are individuals who reveal misconduct, immorality, and wrongdoing within organizations. They are driven by a sense of ethical responsibility, personal beliefs, and a dedication to serving the public good (Dungan et al., 2015). They may belong to different departments within a company, ranging from low-level staff to senior managers. They are usually interested in identifying misconduct in their organizations, and they are ambitious to maintain honesty across all levels (Martin, 2013). As Figure 2 illustrates, they frequently encounter significant challenges, including retaliation, harassment, and job loss. These adverse consequences can deter potential whistleblowers from coming forward. Protection measures are essential to encourage and safeguard these individuals. Protections (e.g., anonymity, internal reporting channels, legal safeguards, internal support systems, and anti-retaliation policies) are critical (Culiberg and Mihelič, 2017; Martin, 2013).

#### 3.2.2 Alleged wrongdoers

An alleged wrongdoer is the individual or group the whistleblower perceives as having committed unethical, illegal, or other acts that led to the whistleblowing. In the context of whistleblowing, these stakeholders are evaluated based on their influence on the process. For instance, Robinson et al. (2012) demonstrated that whistleblowers are less likely to disclose wrongdoing when they believe the wrongdoer is aware of their knowledge of unethical or illegal acts (Robinson et al., 2012). Bergemann and Aven (2023) showed that greater cohesion within a group could deter whistleblowing when the wrongdoer is part of that group (Bergemann and Aven, 2023). As such, alleged wrongdoers are critical stakeholders who may influence the whistleblowing process.

#### 3.2.3 Organizations

A company's management and culture shape the environment for whistleblowing when management commits to integrity and to developing ethical, transparent work procedures (Vandekerckhove, 2006). Alternatively, aggressive or unresponsive managerial attitudes may suppress whistleblowers' efforts (Gao and Brink, 2017). Responsible organizations implement wide-ranging whistleblowing practices by disseminating clear details to those concerned on how to report illegal activities. Such policies must ensure that confidentiality and privacy are always maintained. Reporting channels should be clear and offer protection when the party allegedly responsible for the misconduct seeks retaliation (Culiberg and Mihelič, 2017). Organizations should regularly revise and update their policy frameworks to ensure they remain effective and responsive to whistleblowers' needs (Vandekerckhove, 2006).

### 3.3 Factors and effects

Whistleblowing, as an essential instrument for ensuring accountability within an organization, is shaped by various elements. Cultures that promote ethical behavior and transparency in the workplace are conducive to whistleblowing (Smaili, 2023). Conversely, employees may be dissuaded from reporting malfeasance if their organizations are toxic enough to stifle criticism, indicating the significant role of organizational culture in shaping the whistleblowing process.

The assistance that whistleblowers receive from the legal system and institutions is another important component. According to Skupień (2021), adequate protection laws and reporting mechanisms increase the likelihood that employees will blow the whistle when appropriate, as they feel more secure when the possibility of retaliation is reduced (Skupień, 2021). Whistleblowers often consider the potential personal consequences (which may include losing their job, suffering damage to their professional reputation, and facing legal ramifications (Baraibar-Diez and Odriozola, 2022). Therefore, understanding personal risk is fundamental. In many instances, whistleblowers experience tremendous stress due to these perceived risks, which influences their decision to report malfeasance.

Social circumstances and peer pressure also influence the decision to whistleblow. Their colleagues' views may influence an individual's decision to report misconduct, whether encouraging or discouraging (Lamba, 2022). Those who blow the whistle frequently look to their social groups for approval. Unfavorable reactions among their peers may discourage them from coming forward. People blow the whistle on wrongdoing for various reasons (Kang, 2022). While some are motivated by ethical concerns, others are motivated by personal gain or external demands. To forecast whistleblowers' conduct, it is important to understand these motivations.

Managerial support within a company may either facilitate or inhibit whistleblowing. Leaders who encourage ethical behavior and advocate transparency often cultivate environments where whistleblowers feel comfortable and supported (Singh and Ramdeo, 2020). Similarly, the degree and nature of the wrongdoing may influence the possibility of whistleblowing. More significant infractions tend to elicit stronger responses from individuals (Chen and Zhang, 2025). Furthermore, public media attention may encourage or discourage people from blowing the whistle. In many cases, whistleblowers receive more support as a result of increased media visibility; however, this prominence may also subject them to public scrutiny and retaliation (Okpor and Dikmen, 2021).

Various factors influence the intention to report. Near and Miceli (1985) highlighted whistleblower characteristics (including an internal locus of control, moral judgement, and specific demographics) that influence reporting (Near and Miceli, 1985). Recipient characteristics, including the recipient's position, inquiry level, and channel anonymity, also influence the reporting process. In addition, factors such as power, credibility, and past experience with reporting misconduct influence decisions to report (Gao and Brink, 2017). Table 2 summarizes the determinants of whistleblowing. It encompasses five foundational determinants of whistleblowing: the whistleblower, the report recipient, the wrongdoer, the wrongdoing, and the organization.

**Table 2:** Summary of the whistleblowing process and determinants.

Dimension	Key determinants/mechanisms
Whistleblower	Moral judgment and locus of control shape willingness to report; perceived risks/rewards and prior speak-up experience matter.
Recipient	Authority and independence determine whether a report triggers action; anonymity/confidentiality and feedback build trust.
Wrongdoer	Power, group cohesion, and the reporter's awareness can suppress disclosure; retaliation capacity magnifies perceived cost.
Wrongdoing	Greater severity and clearer harm increase reporting; strong evidence reduces uncertainty; systemic/core-process issues raise stakes.
Organization/culture	Ethical climate and tone at the top enable speaking up; anti-retaliation rules, resources, and a strong handling track record protect reporters.

According to this model, the attributes of the wrongdoing (e.g., organizational dependency and evidence credibility) are highly related to reporting intentions. Organizational characteristics, such as a culture that encourages whistleblowing, an ethical climate, and the organizational structure, also affect the inclination to report. Offering a similar explanation, Lee and Xiao (2018) emphasized factors impacting internal and external whistleblowing by incorporating elements such as whistleblower traits, costs and benefits, organizational aspects, and characteristics of misconduct and wrongdoers (Lee and Xiao, 2018). Table 3 summarizes the factors affecting whistleblowing. These factors are labeled as personal and environmental, with the environment understood in organizational, legal, political, and cultural terms.

**Table 3:** Factors affecting whistleblowing.

Factors affecting whistleblowing	Description
Personal: personality traits	Personality traits including strong principles, moral convictions and attitudes toward money can determine whistleblowing desires (Brennan, 2020). For instance, Machiavellianism, as characterized by the presence of traits like distrust and anxiety toward ethical dilemmas, may influence reporting.
Organizational environment: organizational attitudes	Positive organizational attitudes toward whistleblowing encourage internal reporting (Lee and Xiao, 2018). Meanwhile, perceptions of retaliation and weak governance structures may discourage both internal and external whistleblowing.
Legal environment: perceived protection	Legal safeguards and protection mechanisms for whistleblowers, as found in legislation, significantly affect people's willingness to report misconduct (Lee and Xiao, 2018).
Political environment: governmental support	Whistleblowing may find support in political and governmental initiatives that foster cultures of transparency. Officials who intend to offer whistleblowers strong means of reporting should eliminate the barriers that discourage reporting.
Cultural environment: ethical norms	Cultural factors, especially ethical norms in each society or organization, influence whistleblowing behaviors. Whistleblowing can be facilitated or thwarted by normative societal values concerning honesty, integrity, and accountability (Gao and Brink, 2017).

Studies of whistleblowing reveal both a common understanding and differences in their conclusions (Brennan, 2020). By connecting whistleblowing with how companies are governed, it stressed a complicated and evolving understanding following the Enron scandal. In contrast, Lee and Xiao (2018) focus on factors leading to whistleblowing in accounting situations which were relatively straightforward, identifying traits of the person speaking up, qualities of the one receiving information, characteristics of those committing misconduct, features of the misconduct itself, and factors related to events at the company/workplace in question.

The preliminary examination of related literature identified several broad areas needing closer analysis. First, awareness and protection gaps persist. Many whistleblowing policies have low awareness of reporting channels and inadequate protective measures. This underscores the need for more effective and accessible systems to support whistleblowers throughout the reporting process (Feinstein et al., 2021). Second, there is a need for broader comparative analysis beyond the well-studied UK and USA systems. Including less-studied regions and fostering cross-learning between developed and developing contexts can help distinguish universal design principles from context-specific adaptations. Such insights can strengthen whistleblowing practices globally (Baljija and Min, 2023). Third, empirical evidence across sectors remains uneven, especially in jurisdictions with fragmented regulation or weak enforcement. Evaluating frameworks in these settings would improve global understanding. Fourth, cultural and regulatory influences are underexplored. Cultural norms and regulatory environments shape attitudes toward autonomy, confidentiality, and organizational loyalty. These, in turn, affect system effectiveness and warrant deeper cross-cultural examination. Fifth, the literature often under-addresses practical implementation challenges. These include fear of retaliation, limited regulatory support, and organizational resistance. More research is needed to mitigate these barriers and improve real-world outcomes. Sixth, the applicability of theoretical frameworks on accountability and public reporting across diverse contexts remains underdeveloped. Further work is needed to understand how to adapt these frameworks to organizational settings with differing governance structures and societal norms. Seventh, non-Western perspectives are largely missing, especially in contexts where governance diverges from democratic structures. These perspectives are essential for a more globally inclusive understanding of whistleblowing.

## 4 Methodology and analytical approach

This study is a theory-informed narrative review. It provides a synthesis of research on whistleblowing in the financial sector, utilizing a narrative review approach (Snyder, 2019). The aim is to support conceptual integration across organizational, legal/regulatory, and governance literatures.

### 4.1 Search strategy and sources

The database search process included key academic databases (Web of Science, Scopus, and EBSCO Business Source Complete) and focused on literature published between 2000 and 2025. The set of search terms included various versions of “whistleblowing,” “whistleblower,” and “protected disclosure,” as well as variations of “financial sector,” “banking,” “fraud detection,” and “corporate governance.” The citations of all identified sources were traced for potential additional sources (Booth, 2016). Suggested sources, provided by legal and financial practitioners and regulatory institutions, along with their respective documents, were also considered (Paez, 2017).

Recognizing that non-Western perspectives are often underrepresented in mainstream literature, we intentionally incorporated complementary evidence from emerging and non-Western contexts, where relevant, on the financial sector and regulatory governance.

### 4.2 Eligibility

To improve transparency, we applied explicit relevance criteria. Sources were included if they (a) addressed whistleblowing/protected disclosure substantively, such as reporting channels, retaliation protection, incentives, organizational response, or effectiveness outcomes; (b) related to the financial sector, such as banking, securities/markets, insurance, compliance, and corporate governance, or showed clear conceptual transfer to financial-sector governance; and (c) fell within the 2000–2025 timeframe. We excluded sources if they mentioned whistleblowing only briefly, focused on non-financial sectors without clear transferability, or did not provide sufficient analytical content for synthesis.

### 4.3 Selection process

Records retrieved from database searches were de-duplicated, screened at title and abstract level, and then assessed at full-text level against the criteria above. Because the review was designed as a theory-informed narrative review rather than a protocol-driven systematic review, we do not report PRISMA-style stage-by-stage screening counts.

### 4.4 Quality appraisal and robustness considerations

A formal quality scoring system or standardized critical appraisal checklist was not applied to all included sources. This approach aligns with the review's theory-informed, integrative purpose and the diverse evidence base considered, which includes peer-reviewed empirical studies and reviews, conceptual and theoretical contributions, and selected regulatory or practitioner documents. Since a single appraisal instrument is not readily comparable across these evidence types, explicit relevance criteria and structured screening were used to determine inclusion.

To enhance credibility in the absence of formal scoring, peer-reviewed academic sources were prioritized where available. Regulatory and practitioner documents were included selectively when issued by recognized or competent bodies and when necessary to describe financial-sector governance arrangements, legal frameworks, or implementation context. The jurisdictional and sectoral context was retained when interpreting the findings to avoid overgeneralization beyond the settings discussed in the source material.

### 4.5 Analytical synthesis process

The synthesis followed a theory-informed narrative approach within an integrative review design. Included sources were organized to facilitate comparisons across contexts and the five theoretical lenses applied in this review. For each source, the primary focus (such as reporting channels, retaliation protection, incentives, organizational response, or effectiveness outcomes), the sector and jurisdictional context (where specified), and the emphasized whistleblowing process component (such as motivation or decision, channel choice, organizational response, escalation, or outcomes) were recorded.

The five theoretical perspectives introduced in Section 2 served as interpretive lenses during synthesis and structured the analysis in Section 5. Evidence was examined for concepts relevant to each lens, including individual ethical appraisal and motivation, perceived instrumentality of reporting, organizational power dynamics and response, institutional implementation and enforcement conditions, and networked governance or accountability arrangements. When sources addressed multiple lenses, observations were recorded under each relevant lens to preserve complementary interpretations.

Integration across lenses involved iterative comparison of lens-based observations to identify recurring patterns of convergence and divergence, as well as to note context-specific qualifications. When findings or interpretations were mixed, conclusions were stated conditionally and explicitly linked to contextual factors such as enforcement credibility, reporting infrastructure, organizational culture, and power asymmetries, rather than being presented as universal effects.

This synthesis logic was informed by established guidance on narrative synthesis (Popay et al., 2006), particularly in developing a preliminary synthesis, exploring relationships across sources, and considering robustness, and was adapted here for a theory-informed integrative review.

Table 4 summarizes the evidence-identification routes, relevance-based screening steps, and theory-guided synthesis process used to structure the analysis in Section 5.

**Table 4:** Evidence identification, relevance-based screening, and theory-guided synthesis workflow (2000–2025).

Step	Inputs	Procedure	Basis for inclusion
1. Database search	Web of Science; Scopus; EBSCO Business Source Complete	Searched 2000–2025 using whistleblowing/protected disclosure and financial-sector governance terms.	Substantive relevance to whistleblowing/protected disclosure in financial services (or clear transfer).
2. Supplementary sources	Citation tracing; practitioner/regulatory suggested sources	Traced citations and considered suggested sources/documents from practitioners and regulators.	Same relevance criteria; included selectively when authoritative and needed for governance/legal/implementation context.
3. Screening	Records from steps 1–2	De-duplicated; screened titles/abstracts; assessed full texts against explicit criteria.	Included if substantive whistleblowing content, financial-sector relevance/transfer, and within 2000–2025.
4. Synthesis organization	Included sources	Organized and interpreted evidence using five theoretical lenses to structure section 5.	Incorporated where it supported theory-guided interpretation across individual, organizational, and system/jurisdiction levels.

### 4.6 Illustrative case selection

In addition to the cross-jurisdiction synthesis, we include three widely 457 documented financial-sector scandals (LIBOR, Danske Bank, and Wirecard) as illustrative mechanism 458 cases. These cases were purposively selected from the included evidence base because they (a) involve 459 material misconduct in regulated financial markets with cross-border implications; (b) have publicly 460 documented disclosure/whistleblowing dynamics and organizational/regulatory responses; and (c) 461 collectively highlight distinct mechanisms aligned with our analytic framework (e.g., organizational 462 power dynamics and retaliation risk; moral commitment and perceived instrumentality of reporting; 463 institutional enforcement credibility; and cross-border supervisory coordination). The purpose of these 464 cases is explanatory.

## 5 Results and discussion

Section 5 reports synthesized patterns identified across the reviewed literature, organized using the five theoretical lenses and key process components. Practical implications and design considerations are consolidated in Section 7 and are presented as interpretive inferences rather than definitive causal claims.

### 5.1 The financial sector as a governance case study

The financial sector provides a particularly relevant context for studies of whistleblowing. ACFE datasets routinely place banking/financial services high volumes of occupational fraud cases. Fraud schemes in the industry often involve corruption, the misappropriation of assets, and the fabrication of financial statements (ACFE, 2024). The prevalence of such misconduct underscores the importance of whistleblower policies in deterring potential fraud (Belgacem, 2025). Association of Certified Fraud Examiners **(**ACFE) data show that tips detect 43% of occupational fraud, about three times any other method, followed by internal audit (14%) and management review (13%), which together account for 70% of cases; 52% of tips come from employees and roughly one-third from external parties, and organizations with hotlines are nearly twice as likely to detect fraud via tip as those without (ACFE, 2024). In line with these detection patterns, whistleblowing has been shown to save organizations millions of dollars annually by reducing both the size and duration of fraud schemes. Despite its effectiveness, retaliation remains a persistent barrier to reporting, particularly where protection and follow-through are perceived as weak (OECD, 2016, 2022). Complementing this, cross-sector studies also reveal differences in reporting behavior. Public sector employees report at higher rates when robust legal protections are in place (Terracol, 2018), whereas in financial services, hierarchical cultures and reputational risks lower the likelihood of disclosure (Chalouat et al., 2019). Moreover, in emerging economies, underreporting is common: FATF (2007) notes that weak enforcement and cultural disincentives contribute to the persistence of money laundering and corruption risks.

Moral judgment and expectancy theories elucidate why the structure of the financial sector can intensify the perceived cost–benefit analysis associated with disclosure. Moral judgment theory posits that individuals are more likely to report wrongdoing when moral motivation surpasses perceived personal risk (Dungan et al., 2019). In financial services, perceived risk is heightened by authority gradients, technical complexity, and uncertainty regarding enforcement, which can suppress internal reporting even when moral concern is significant (Batolas et al., 2023). Expectancy theory further suggests that whistleblowing is less likely when individuals perceive low instrumentality, meaning that reporting is not expected to result in corrective action or protection, particularly in contexts with limited enforcement visibility (Potipiroon, 2024).

This section utilizes the theoretical models developed in Section 2.2 to examine the specifics of whistleblowing in the finance industry. We illustrate how institutional theory explains cross-national differences in the effectiveness of reporting, how power-dependence theory accounts for organizational responses to disclosure, and how network governance frameworks elucidate the role of cross-border regulatory systems. Through an appraisal of sector-specific risk, the regulatory framework, case notoriety, and comparative evidence from Western and non-Western contexts, we demonstrate that whistleblowing outcomes in finance result from a complex interplay of personal moral judgment, organizational power dynamics, institutional constraints, and transnational regulatory networks. This holistic treatment outlines both systemic vulnerabilities that necessitate whistleblowing in finance and the institutional leverage points where policy can have the most impact.

Beyond this vulnerability, the need to study whistleblowing in the financial sector arises from the sector's systemic importance to the global economy. This importance was evident in the global economy when interconnected banking systems contributed to the spread of contagion across multiple countries (Brunnermeier, 2009). However, the literature on whistleblowing in the sector highlights the absence of uniform, sector-wide practices. While regulations such as the Sarbanes–Oxley Act of 2002, the Dodd–Frank Act of 2010, and directives in the European Union have expanded whistleblower protections in the West (Bigus and Momsen, 2023; European Parliament and Council of the European Union, 2019; U.S. Congress, 2010; U.S. Department of Labor, 2002). In many emerging economies, comprehensive whistleblowing frameworks are still developing. Reforms in Qatar's financial sector represent important progress, with further scope to enhance implementation and embed protections more consistently across institutions.

### 5.2 Cumulative sectoral risks and structural vulnerabilities

Across the reviewed literature, fraud exposure in financial services is commonly discussed as arising from sectoral risks and structural vulnerabilities, which, in turn, motivate attention to whistleblowing and integrity mechanisms. In parts of the Gulf region, many firms may still be developing structured reporting systems for ethical practices (Safari et al., 2022). The limited uptake of such systems reflects evolving governance frameworks, which continue to mature as institutions strengthen their compliance capacity. Related work also notes that banking institutions operate in political and cultural environments that may shape the implementation of internal whistleblowing mechanisms in practice (Ahmed and Mohamed, 2025).

At the same time, the sector faces pressures linked to technological change. Greater reliance on digital channels, third-party providers, and FinTech-enabled services can increase operational and cyber risk exposure, which may expand opportunities for fraud and misconduct (Basel Committee on Banking Supervision, 2018; International Monetary Fund, 2024). Regulators therefore emphasize that governance and control frameworks should evolve with innovation, including confidential speak-up/whistleblowing arrangements to surface concerns early; where such protections are weak, under-reporting can delay detection and, in severe cases, carry broader financial-stability implications (Basel Committee on Banking Supervision, 2015; International Monetary Fund, 2024).

Interpreted through the institutional and expectancy lenses, these accounts are commonly used to highlight how governance maturity and perceived instrumentality of reporting may condition the credibility and uptake of whistleblowing mechanisms.

### 5.3 Regulatory frameworks and sectoral enforcement

Although various frameworks have been developed worldwide to facilitate whistleblowing, these frameworks are not universally adopted. In the United States (US), for instance, regulations such as SOX and Dodd–Frank have emphasized the establishment of corporate governance environments that support whistleblowing in publicly traded securities. The primary analytical framework for understanding the observed regulatory differences is institutional theory. It explains how the interplay among formal rules, informal norms, and societal expectations creates distinct modes of organizational behavior across jurisdictions. In jurisdictions where regulatory enforcement is credible and cultural norms promote transparency (as is the case in mature Western systems), whistleblowing mechanisms are linked to organizational practices. In jurisdictions where enforcement is weak or hierarchical norms deter questioning of authority, formal constructs are present but provide little, if any, substance, a pattern evident in transitional economies. In the European Union, for example, the EU Whistleblower Directive (first implemented in 2019) provides an important framework for strengthening protections across member states (Vandekerckhove et al., 2023).

As a policy framework, the EU Whistleblower Directive sets minimum standards for protected channels and confidentiality. The European Banking Authority (EBA) Guidelines on Internal Governance require banks to maintain credible internal reporting/whistleblowing arrangements (European Union, 2019; EBA, 2021). To strengthen cross-jurisdictional comparison and reduce repetition, Table 5 synthesizes how the main regulatory approaches discussed in Sections 5.3 and 5.5 map onto the theoretical mechanisms developed in Section 2.2. In brief, regimes tend to be more effective when formal protections are paired with reliable enforcement and supervisory follow-through (institutional theory), when reporting channels reduce organizational control over outcomes (power–dependence theory), and when incentives increase the perceived instrumentality and valence of reporting (expectancy theory). Where rules exist but enforcement and organizational uptake are weak, compliance can remain largely symbolic, and reporting is discouraged or displaced to external escalation.

**Table 5:** Cross-jurisdiction comparison and theory-linked mechanisms (structured narrative synthesis from sections 5.3 and 5.5).

Jurisdictional category	Key feature	Implementation emphasis	Primary theory lens
US/EU	Major whistleblowing frameworks in finance (SOX/Dodd–Frank; EU Whistleblower Directive)	Mature institutional context: EU effectiveness depends on transposition and enforcement	Institutional; network governance (EU)
Qatar/UAE	Dual-perimeter or sectoral instruments (QFC mechanisms; sectoral codes)	Multi-perimeter regulatory arrangements: uptake linked to the consistency of protections and supervisory follow-through	Institutional; network governance; power–dependence
Malaysia/India	Statutory protections (2010/2014 acts)	Described as uneven/limited impact in practice (awareness and implementation challenges)	Institutional
Cross-cutting (all contexts)	Organizational dynamics shape reporting	Perceived retaliation risk and control over channels can suppress reporting even where rules exist	Power–dependence; expectancy; moral judgement

In many emerging economies, including Qatar, regulatory approaches to whistleblowing continue to evolve. While sectoral measures are in place, broader whistleblowing frameworks are consolidating and institutionalizing as governance systems mature.

Consistent with the organizational dynamics discussed in Section 5.2, perceived retaliation risk and hierarchical structures continue to shape reporting behavior, particularly where enforcement visibility is limited (Kampourakis, 2021). Ongoing reforms in Qatar's financial sector demonstrate growing recognition of whistleblowing as a governance safeguard, with opportunities to further embed protections and increase awareness at both organizational and sectoral levels.

A comparative analysis of Qatar reveals three key opportunities for future development. First, expanding Qatar Financial Centre (QFC) whistleblower protections to financial firms operating under non-QFC regulatory frameworks would enhance certainty in the financial sector. The QFC Regulatory Authority mandates that authorized firms provide internal reporting channels with confidentiality protections; extending these requirements sector-wide, in alignment with Qatar Central Bank regulations, would reduce regulatory arbitrage and strengthen deterrence. Second, establishing visible enforcement with public outcomes of case determinations would foster a cultural understanding of whistleblowing. When the community observes that disclosures result in substantive investigations and appropriate protections, cultural assumptions about reporting are recalibrated. Achieving this requires both legislative authority and institutional commitment from regulators to act on credible disclosures. Third, incorporating whistleblower measures into Basel Pillar 2 supervisory reviews would create institutional incentives for banks to enhance their reporting mechanisms. Banks would be evaluated on the effectiveness of their reporting channels, response timeliness, and absence of retaliation, thereby embedding whistleblowing incentives within the operational risk management framework established in regulatory practice.

From a broader perspective, however, differences in the enforcement of whistleblower regulations across countries persist, leaving vulnerabilities within the global financial system. The Basel framework provides one avenue for harmonizing international standards. Banks with significant global exposure are required to evaluate their operational risks, including fraud (Basel Committee on Banking Supervision, 2025). This obligation may encourage the development of stronger whistleblower protections even in jurisdictions where frameworks are still emerging. Similarly, the extraterritorial reach of US laws, such as the Foreign Corrupt Practices Act, has influenced non-US financial institutions to adopt stronger compliance mechanisms (Karpacheva and Hock, 2024).

### 5.4 Case-based reflections: from LIBOR to Wirecard

Cases of fraud in the financial sector illustrate the sector's acute need for whistleblowing. When fraudulent activities are discovered within banking organizations, they often result in large-scale institutional failures, as demonstrated by the LIBOR manipulation, Danske Bank's money-laundering scandal, and the collapse of Wirecard. These cases had significant ramifications for the global economy and exposed weaknesses in internal reporting and governance systems. These three cases are used as illustrative examples (not an exhaustive set) because each is well-documented, cross-jurisdictional, and highlights a different point of governance failure, benchmark setting (LIBOR), anti–money laundering controls (Danske Bank), and financial reporting/audit oversight (Wirecard), allowing clearer mapping to the mechanisms in our multi-theoretical framework.

The LIBOR scandal, for example, highlights how deterrents to whistleblowing persist even in highly regulated markets. Some individuals who raised concerns faced sanctions or prosecution, underscoring the risks that discourage potential whistleblowers from coming forward (Verity, 2022). The Wirecard case further demonstrates the importance of social and familial support in enabling disclosure. Here, the whistleblower was sustained by personal networks, which alleviated psychological pressures that might otherwise have silenced reporting (McCrum et al., 2021). Similarly, Danske Bank demonstrates the decisive influence of organizational culture and leadership: external reporting was only chosen after internal channels failed to act on suspicious transactions (Reuters, 2018).

Taken together, these cases demonstrate that even in jurisdictions with advanced legal frameworks, internal governance failures can drive misconduct underground until external whistleblowing exposes it. They also illustrate how personal, organizational, and cultural factors intersect with regulatory protections to shape whistleblowing outcomes. Utilizing the theories from section 2.2, each case study presents a distinct mechanism pattern. The LIBOR scandal illustrates power–dependence theory. Whistleblowers attempted to influence a dominant coalition and encountered retaliation. LIBOR also reflects an expectancy mechanism: perceived low protection and low instrumentality of internal escalation can suppress reporting, even in highly regulated markets. The Wirecard case illustrates moral judgment and social connection. Here, the whistleblower persisted in disclosing despite personal and professional costs. This case highlights an expectancy mechanism shaped by beliefs about whether external audiences, such as media or regulators, will respond credibly. The Danske Bank case exemplifies institutional theory. Internal disclosures were ineffective under organizational norms prioritizing clients and profits over compliance, leading to external escalation. It also highlights a network-governance mechanism, given cross-border anti–money laundering supervision and the need for coordination across jurisdictions. Together, these cases show that whistleblowing effectiveness in finance depends on a supportive context across multiple levels: individual (moral commitment and perceived instrumentality), organizational (power dynamics, culture, and accountability), and institutional/regulatory systems (reliable enforcement and cross-jurisdictional coordination).

### 5.5 Non-Western case insights

The illustrative non-Western contexts discussed in this subsection (including Qatar and Singapore) are included to examine how the mechanisms identified in the reviewed literature may operate under different enforcement and cultural conditions.

Outside the Western context, reforms often highlight the challenges of translating statutory protections into high-trust reporting environments. Malaysia's Whistleblower Protection Act (2010) demonstrates the limits of reform without reliable enforcement, as reporting levels remain low and awareness is limited (Leong, 2017; Ramli et al., 2018). India's Satyam scandal similarly exposed weaknesses in corporate governance and contributed to the adoption of the Whistleblower Protection Act, 2014 (Press Information Bureau Government of India, 2024). In the Gulf, jurisdictions such as the UAE have introduced sectoral codes of conduct, while Qatar continues to strengthen financial-sector reforms and expand cultural and institutional support for whistleblowing (Safari et al., 2022; Chalouat et al., 2019). Collectively, these cases contrast with the US/EU experience in Section 5.3, where formal protections are more routinely coupled with enforcement capacity and supervisory follow-through, shaping greater confidence that reporting will be acted on.

Institutional theory explains these disparate results by emphasizing the credibility of enforcement and cultural fit. Malaysia and India illustrate how formal laws, in the absence of visible enforcement, can produce symbolic compliance rather than behavioral change, as pressures to protect the organization outweigh pressures for transparency. Qatar presents a more hybrid institutional configuration. The Qatar Financial Centre (QFC), established in 2005, operates within an independent regulatory environment under the supervision of the QFC Regulatory Authority (QFCRA) (QFC, 2025a,b). Within the QFC, QFC-authorized firms are required to maintain internal mechanisms to protect reporters; beyond the QFC perimeter, regulatory coverage and enforcement arrangements follow the applicable sectoral framework (QFC, 2017, 2025c). This dual structure is analytically important: power-dependence dynamics inside firms may remain salient where internal channels are perceived as controllable, while institutional credibility can be strengthened by widening the reach of QFC-aligned requirements, communicating protections, and demonstrating follow-through through visible enforcement actions.

By contrast, Singapore represents a regional best practice. Its Corrupt Practices Investigation Bureau (CPIB) has established secure and anonymous whistleblowing channels, while the Monetary Authority of Singapore (MAS) requires financial institutions to implement robust internal reporting systems. These mechanisms are supported by strong political commitment to integrity, a culture of compliance, and explicit legal protections, which together encourage disclosure and safeguard whistleblowers (Quah, 2018). Singapore's model demonstrates how combining legal enforcement with institutional credibility and cultural support can create an environment in which whistleblowing is both effective and sustainable.

## 6 Limitations

In this review, we identified several limitations related to the methodology and scope. First, the narrative literature review is primarily based on English-language publications. As a result, region-specific scholarship and practice-based evidence may be underrepresented. This language and geographic concentration may limit the extent to which findings can be generalized globally. Second, the available literature may be subject to selection and reporting biases, as it often emphasizes whistleblowing cases with visible outcomes or public documentation. Less visible experiences, such as concerns that were not recorded, did not progress, or remained confidential, may therefore be underrepresented, skewing the evidence base toward higher-profile cases. Third, empirical data available to verify the effectiveness of whistleblowing in transitional (or emerging) economies is sparse, necessitating reliance on regulatory documents, policy studies, and documented cases rather than outcome studies with systematic designs.

Given that the evidence base includes secondary sources such as regulatory documents, policy reports, and documented cases, the policy implications are presented as practical governance and design principles rather than definitive causal claims. We therefore highlight key transfer conditions (reliable enforcement, confidentiality protections, institutional independence, and cultural fit) and recommend piloting and monitoring reforms before scaling them.

The development of institutional theory helps explain these differences across national contexts. However, we need more empirical attention to the finer-grained aspects of investigations into theoretical predictions by examining actual reporting and organizational response patterns across cultures. Finally, rapidly advancing technology is driving blockchain-based anonymous reporting and AI-assisted fraud detection. The observations in the current review will quickly become obsolete by new modes of operation changing those merely “opportunity” or “risk” disclosure processes.

## 7 Implications for culture, compliance, and reforms

The reviewed literature highlights the importance of organizational culture and ethical leadership in developing strong whistleblower mechanisms within an organization. For instance, while moral judgment theory suggests that some whistleblowers may be motivated by moral concern, other theories propose that whistleblowing involves considering personal costs and benefits. In this respect, an ethical organizational culture can support the establishment of robust whistleblower mechanisms by discouraging retaliation against whistleblowers. Moreover, since those charged with governance are often the ones to whom reports of malfeasance are made, ethical leadership is crucial in ensuring the credibility of reporting mechanisms. As the literature illustrates, absent a credible reporting system, organizations are unlikely to benefit from the envisaged role of whistleblowing in deterring occupational fraud.

Specifically for the banking sector, the reviewed literature highlights the need for collaboration in developing and enforcing whistleblower protections worldwide. The lack of uniform standards has contributed to variations in reporting across emerging economies, including Qatar, where ongoing reforms are gradually addressing cultural and organizational factors that have historically influenced reporting behavior. In this context, strengthening corporate governance arrangements to include credible whistleblower provisions is often framed as an opportunity aligned with diversification and investment objectives. Where protections are credible and adopted in practice, they may support investor confidence, reduce operational risk exposure, and strengthen perceptions of financial-sector integrity in international markets. Nevertheless, due to the interconnectedness of the global financial system, fraud in banks in one country can spread across the system. Widespread whistleblower protection standards, informed by guidance already established in developed economies, may help reduce the risk of fraudulent activity when matched by credible enforcement, organizational follow-through, and regular monitoring of reporting and retaliation outcomes, thereby supporting banking system stability.

While the core features of whistleblowing systems are often similar (confidential intake, protection against retaliation, documented follow-through), implementation can vary with institutional capacity and resource availability. In higher-capacity contexts, regulators and large institutions may have scope to introduce more comprehensive arrangements. For example, they might add independent reporting options, more formal case-tracking systems, dedicated investigative capabilities, and periodic disclosure of aggregated outcome indicators. These indicators could include time to first review, confirmation rates, corrective actions, and retaliation findings. In transitional or emerging economies, a phased approach is often more feasible. This can begin with clear protected channels and consistent intake and escalation protocols, then build investigation capability, oversight, and monitoring over time. In more resource-constrained settings, practical measures may focus on low-cost options. Examples include simple anonymous reporting mechanisms, basic confidentiality safeguards, trained focal points, and proportionate consequences for retaliation. These are supported by awareness efforts that clarify how reports are handled and what protections apply. Across contexts, piloting and periodic review can help assess uptake, trust, and outcomes before wider scaling.

On the other hand, recent research indicates that whistleblowing can be experienced as an “intensive trajectory” for some individuals, with implications that extend beyond formal procedures, although evidence remains context-dependent and varies across sectors (Van Eijbergen and Siebers, 2025). A recent scoping review in a healthcare reporting context maps the psychosocial implications discussed in the literature and highlights areas where evidence remains limited, reinforcing that the “human” experience may not be fully captured by policy or compliance lenses alone (McDonald et al., 2024). Qualitative and narrative work also describes how reporting can intersect with workplace belonging and identity, including accounts of identity strain and social dislocation in policing settings (Stubbs and Tong, 2025). In addition, communication research suggests that, in some cases, the consequences of whistleblowing can spill over into family and social identity (Richardson, 2023).

## 8 Current trends and future directions

Current trends include the development of various institutions to improve whistleblowing mechanisms in both the public and private sectors. Loyens and Vandekerckhove (2018) noted an increase in government agencies dealing with whistleblowing aspects in the 11 countries examined. These agencies address various aspects of whistleblowing, including investigations into retaliation, assessments of wrongdoing, and the development of policies to prevent such misconduct (Loyens and Vandekerckhove, 2018). Another observed trend is the increasing application of US whistleblower laws worldwide. Enforcement of the Foreign Corrupt Practices Act for both the US and non-US corporations implicated in economic crime has influenced the safeguards that organizations implement to mitigate the risk of such fraud, including bribery (Karpacheva and Hock, 2024). Accordingly, the increased extraterritorial application of the US anticorruption laws has affected the development of whistleblowing mechanisms in diverse countries.

Future directions include developing global perspectives on whistleblowing and understanding the impact of emerging technologies, such as social media, on the whistleblowing process. There has been limited examination of the influence of cultural context on whistleblowing; most studies focus on individual, organizational, and legislative effects (Supendi et al., 2023). Cultural context may nevertheless impact whistleblowing, as collectivistic cultures or culturally conservative environments can act as barriers to whistleblowing by fostering the perception that such acts constitute disloyalty. Moreover, as technologies, including social media, affect how individuals interact, they may influence whistleblowers' decisions—especially in cases of external whistleblowing. These technologies have enabled the rapid spread of information, leaving organizations with short time frames to respond to allegations of wrongdoing when they are subject to external whistleblowing. Another technology that could enhance whistleblowing is blockchain, which, as recent research demonstrates, can be used to develop channels that facilitate anonymous reporting and thereby alleviate underreporting due to fear of retaliation (Mbimbi et al., 2025). Moreover, it has been suggested that generative AI could help employees identify wrongdoing and report it (Dimitrios, 2019). Additional prospects include using AI to report cases of wrongdoing without human intervention. However, such implementations may be problematic – especially in the financial sector, where issues of data privacy and confidentiality are prioritized.

## 9 Conclusions

Whistleblowing has become a core mechanism for detecting corporate malfeasance, especially in environments where organizations lose significant amounts to occupational fraud. The contribution of this review is to bring together many theoretical perspectives (moral judgement, power-dependence, expectancy, institutional, and network governance theories) to illustrate how whistleblowing in the financial sector is a multifaceted phenomenon. The review does not confront these theories as rival explanations but rather integrates their insights: moral judgement explains why individuals report despite risking personal consequences, power-dependence shows how organizations respond and retaliate, expectancy theory explains how incentives and protection structure reporting decisions, institutional theory explains how cross-national differences in effectiveness arise, and network governance shows how transnational standards can create accountability mechanisms that extend beyond national venues. The literature highlights power-dependence, expectancy, and moral judgment theories to explain the whistleblowing process and guide the development of stronger protections that deter occupational fraud. Across jurisdictions, approaches vary. In emerging economies, including Qatar, Malaysia, and India, whistleblowing frameworks are at different stages of development, with sectoral reforms in place and ongoing efforts focused on consolidation and consistent implementation. Cultural and organizational factors, such as institutional structures and confidentiality considerations, influence how these frameworks are used in practice. Including these contexts and noting Singapore as a regional best practice helps redress a Western-centric literature and supports globally inclusive designs. Policy-wise, robust protections in the US and EU have incentivized organizations to embed whistleblowing in their governance. Aligning with Basel operational risk requirements offers a practical path for global financial governance. Given its exposure to fraud and cross-border links, the financial sector stands to gain the most from such harmonization.
